# Acknowledgement to Reviewers of *Metabolites* in 2013

**DOI:** 10.3390/metabo4010129

**Published:** 2014-02-26

**Authors:** Metabolites Editorial Office

**Affiliations:** MDPI AG, Klybeckstrasse 64, CH-4057 Basel, Switzerland

The editors of *Metabolites* would like to express their sincere gratitude to the following reviewers for assessing manuscripts in 2013:
Åberg, K. MagnusHenry-Kirk, Rebecca A.Ovenden, SimonAlber, Birgit E.Hihara, YukakoPaik, Man-JeongAlfermann, A. W.Hildebrand, MarkPakhomova, OlgaAllen, Doug K.Hill, Herbert H.Lopez, Pascal J.Amann, AntonHiller, KarstenPetersen, MaikeAprea, EugenioHirst, JenniferPiccolo, AlessandroBain, JamesHorning, StevanPluskal, TomášBaracos, Vickie E.Horst, IrmtraudPutluri, NagireddyBarlow, ChrisHoury, Walid A.Rabinowitz, JoshuaBaumbach, Joerg IngoHouten, Sander MichelRandell, EdwardBearden, DanHsieh, Ming-faReijmers, TheoBeckles, Diane M.Jiang, FengReinfelder, John R.Beisken, StephanJuergen, SchmidtRichards, SelenaBettenbrock, KatjaKaddoumi, AmalRichardson, AdamBielawski, J.Kadokawa, Jun-ichiRichardson, SusanBlasco, HélèneKataoka, H.Rokitta, Sebastian D.Böcker, SebastianKempa, StefanRomance, MiguelBodvard, KKhoomrung, SakdaSaha, AsishBoros, LaszloKikuchi, JunSakai, AtsushiBraun, Ralf J.Kind, TobiasSakellariou, DimitrisBruni, PaolaKremling, AndreasSalazar, Eduardo PerezBurgess, Karl E. V.Krömer, Jens OSalehi-Ashtiani, KouroshCardinali, GianluigiKuchel, PhilipSanchez, Diego H.Cardoso, Vicelma L.Lachenmeier, Dirk W.Scano, PaolaCascio, MichaelLalk, MichaelSchellhorn, Herb E.Causeur, DavidLaluce, C.Schirra, HorstColinge, JacquesLamanna, RaffaeleSchnackenberg, LauraCornett, ClausLang, ChristineSheedy, JohnCreek, DarrenLangley, G. JohnSteinhauser, DirkCzech, ChristianLankhorst, PeterSwindell, William RDandekar, ThomasLannig, GiselaTaamalli, AmeniDavies, ChristopherLarive, CynthiaTani, ShujiDe Figueiredo, Luiś F.Larsen, FlemmingTanokura, MasaruDe Magalhães, João PedroLee, KyongbumTeng, QuincyDe Souza, DavidLegido-Quigley, CristinaThakur, MathewDel Pozo, FranciscoLiebeke, ManuelTikunov, Y. M.Dismukes, G. CharlesLocasale, Jason W.Tonon, T.Duan, Chang QinLuchinat, ClaudioTrygg, JohanDunn, WarwickLusczek, Elizabeth R.Tsai, Tung-HuEnomoto, ToshikiMani, SridharUrayama, ShiroEvans, Anne M.Marchan, R.Van Der Hooft, JustinFendt, Sarah-MariaMarincola, Flaminia CesareVetter, Stefan W.Fernie, AlisdairMarkuszewski, Michał JanVieth, ReinholdFerreira, DanielaMarriott, Philip J.Villas-Boas, SilasFlorencio, Francisco J.Martín, Juan F.Von Kamp, AxelForcisi, SaraMartin, William F.Vuckovic, DajanaFujimura, YoshinoriMercado, Jesús M.Wang, GangGardiner, R. A.Miller, GadWant, Elizabeth JGaunitz, FrankMorris, John HWatt, PeterGeiszler, PhilippineMorvan, DanielWeingart, GeorgGika, H.Motta, AndreaWendell, Stacy GelhausGlenn, Thomas C.Moulisova, VladimiraWesterhuis, Johan A.Goen, ThomasLe Moyec, L.Wilson, Julie C.Gooley, PaulNaidu, RaviYamaji, RyoichiGoto-Yamamoto, N.Nash, DavidYamashita, MichiakiGronwald, WolframNeumann, SteffenYáñez, JaimeGu, HelenNiederman, Prof. RobertYetukuri, LaxmanGulbahce, NataliNishioka, TakaakiYeung, Pollen K.f.Günther, U. L.Nunnery, JoshawnaYin, HuiyongHaick, HossamOberacher, HerbertYoung, JameyHay, JordanOdahara, TakayukiZilliges, YvonneHeckman-Stoddard, Brandy M.Okada, TaketoZou, ZhongmeiiHeizmann, SilkeOno, Eiichiro


